# CpG Usage in RNA Viruses: Data and Hypotheses

**DOI:** 10.1371/journal.pone.0074109

**Published:** 2013-09-23

**Authors:** Xiaofei Cheng, Nasar Virk, Wei Chen, Shuqin Ji, Shuxian Ji, Yuqiang Sun, Xiaoyun Wu

**Affiliations:** 1 College of Life and Environmental Science, Hangzhou Normal University, Hangzhou, Zhejiang, P.R. China; 2 Atta-ur-Rahman School of Applied Biosciences, National University of Sciences and Technology, Islamabad, Pakistan; 3 College of Agricultural and Food Science, Zhejiang Agricultural and Forestry University, Linan, Zhejiang, P.R. China; 4 School of Economic and Management, Zhejiang University of Science and Technology, Hangzhou, Zhejiang, P.R. China; Albert Einstein College of Medicine, United States of America

## Abstract

CpG repression in RNA viruses has been known for decades, but a reasonable explanation has not yet been proposed to explain this phenomenon. In this study, we calculated the CpG odds ratio of all RNA viruses that have available genome sequences and analyzed the correlation with their genome polarity, base composition, synonymous codon usage, phylogenetic relationship, and host. The results indicated that the viral base composition, synonymous codon usage and host selection were the dominant factors that determined the CpG bias in RNA viruses. CpG usage variation between the different viral groups was caused by different combinations of these pressures, which also differed from each other in strength. The consistent under-representation of CpG usage in −ssRNA viruses is determined predominantly by base composition, which may be a consequence of the U/A preferred mutation bias of −ssRNA viruses, whereas the CpG usage of +ssRNA viruses is affected greatly by their hosts. As a result, most +ssRNA viruses mimic their hosts' CpG usage. Unbiased CpG usage in dsRNA viruses is most likely a result of their dsRNA genome, which allows the viruses to escape from the host-driven CpG elimination pressure. CpG was under-represented in all reverse-transcribing viruses (RT viruses), suggesting that DNA methylation is an important factor affecting the CpG usage of retroviruses. However, vertebrate-infecting RT viruses may also suffer host' CpG elimination pressure that also acts on +ssRNA viruses, which results in further under-representation of CpG in the vertebrate-infecting RT viruses.

## Introduction

The relative abundance of neighbor nucleotides (dinucleotides) has been known as a genome signature of species since the 1960s and has been studied extensively in genomic DNA samples from many organisms, including vertebrates, invertebrates, plants, and prokaryotes [1]–[4]. These studies have demonstrated that TpA is under-represented in almost all organisms tested, whereas CpG is differentially represented in the genomes of eukaryotic organisms [2]. TpA depletion is believed to be caused by its presence in two out of three canonical stop codons and in transcriptional regulatory motifs (e.g., the TATA box sequence). Therefore, TpA avoidance reduces the risk of nonsense mutations and minimizes improper transcription [5]. CpG under-representation has been directly linked to cytosine DNA methylation, an epigenetic modification that plays important roles in diverse biological processes, such as gene and transposon silencing, genetic imprinting and X chromosome inactivation [6]. Methylated cytosines are prone to mutate into thymines through spontaneous deamination, resulting in the dinucleotide TpG and the subsequent presence of a CpA on the opposite strand after DNA replication [7]. This result is consistent with the concomitant CpA and TpG over-representation in CpG-suppressed organisms. Thus, the over-representation of CpA and TpG is considered to be a consequence of the under-representation of CpG.

Interestingly, CpG has also been observed to be predominantly under-represented in RNA viruses (in both retroviruses and riboviruses) [8], [9]. CpG deficiency in retroviruses may be due to host cytosine methylation [10], [11]. However, the precise mechanism that contributes to CpG under-representation in riboviruses is still largely unknown. Because riboviruses do not form DNA intermediates during genome replication, the methylation-deamination model is unlikely to apply. To date, two non-exclusive explanations have been suggested to explain the prevalence of CpG under-representation in riboviruses: the nucleotide-stacking energy model and the host innate immunity evasion model. The nucleotide-stacking energy model is based on the fact that in DNA duplexes, CpG has a much higher stacking energy than other dinucleotides, which may reduce the rate of transcription and replication of viruses [12]. However, this hypothesis has been challenged by the subsequent finding that the free energy of RNA duplexes of CpGs lies in the middle of all 16 possible dinucleotides [13].

The second hypothesis is based on the observation that the CpG odds ratio values of mammal-infecting riboviruses are lower than the riboviruses infecting other taxa. Influenza A virus, which originated from an avian reservoir, has undergone significant CpG reduction since its introduction into humans [14]. This hypothesis is further reinforced by the fact that the CpG motif in an AU-rich oligonucleotide can significantly stimulate the immune response of plasmacytoid dendritic cells [15], [16]. Furthermore, the replicative fitness of poliovirus decreases sharply with increased frequencies of the dinucleotides CpG and UpA in the capsid region [17]. Nevertheless, this hypothesis cannot explain the normal distribution of CpG in riboviruses infecting other taxa, such as invertebrates [18] and the under-representation of CpG in some plant viruses [8], [9]. In this study, we examined CpG usage in RNA viruses and their hosts to further address CpG under-representation in RNA viruses.

## Results

### Data characterization

We downloaded all available full genomic sequences of RNA viruses [including both riboviruses and reverse-transcribing viruses (RT viruses)] from the RefSeq database. After removing inaccurate sequences, a total of 1,955 sequences was obtained, which represented the genome of 1120 RNA viruses. These viruses belong to 61 different viral families or unassigned genera, which include 95.3% of the recognized viral families or unassigned genera from the Ninth Report of the International Committee on Taxonomy of Viruses (ICTV) (Dataset S1). The downloaded genome sequences covering virtually all types of RNA viruses identified thus far, as well as the host categories that they infect.

### Overall CpG variation in RNA viruses

To obtain an overall view of CpG usage in RNA viruses, we examined the CpG odds ratio, the observed CpG incidence normalized to the expected CpG frequency (CpG*_O/E_*), of each viral genome (Dataset S2). When there is no selection (i.e., when all 16 dinucleotide pairs are randomly used), the CpG frequency should be similar to its expected frequency, and the CpG*_O/E_* value should approach 1. A CpG*_O/E_* value of a virus ≤0.78 or ≥1.23 indicates that it is significantly under-represented or over-represented in that virus [19]. Accordingly, a CpG*_O/E_* value between 0.79–1.12 can be recognized as being in the normal frequency range. The mean CpG*_O/E_* value of these RNA viruses was 0.67±0.240 (Table 1), with 0.15 and 1.35 as the minimum and maximum CpG*_O/E_* values, respectively. CpG was found to be significantly under-represented in 744 RNA viruses and over-represented in 12 RNA viruses, whereas it was normally distributed in the rest 364 viruses. These results suggest that CpG is under-represented in most of RNA viruses examined.

**Table 1:** CpG usage of RNA viruses.

	CpG*_O/E_*	CpG*_O/E_*__CDS_	CpG*_O/E_*__CDS_12_	CpG*_O/E_*__CDS_23_	CpG*_O/E_*__CDS_31_
Mean odd ratio	0.67±0.240	0.65±0.248	0.50±0.214	0.58±0.266	0.90±0.395
CpG*_O/E_* ≤0.78	744	749	1019	879	489
CpG*_O/E_* 0.79–1.22	364	358	101	221	394
CpG*_O/E_* ≥1.23	12	13	0	20	237

**Figure 1:**
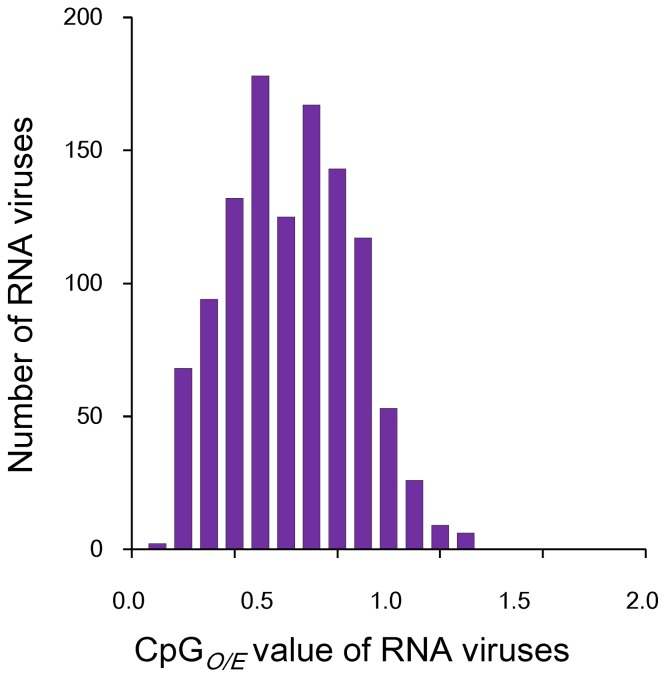
CpG usage pattern of RNA viruses. The *y*-axis depicts the number of viruses with the specific CpG*_O/E_* values given on the *x*-axis.

To obtain further insight into the variation of CpG bias in RNA viruses, we produced a CpG*_O/E_* distribution profile (Figure 1). The CpG*_O/E_* distribution profile deviated negatively from the normal range, which also suggests that CpG is under-represented in most of the examined RNA viruses. Instead of a unimodal pattern, the CpG distribution profile appears to be composed of more than one normal distribution. To test whether this special distribution pattern is unique to CpG, we further analyzed the distribution patterns of the other 15 dinucleotides (Figure S1). The distribution patterns of the other 15 dinucleotides displayed an apparently unimodal distribution pattern (Figure S1). In addition, the distribution range of CpG (ranging from 0.15 to 1.35) was broader than that of other dinucleotides. These results suggest huge variations of CpG bias in RNA viruses.

### Effect of base composition

**Figure 2:**
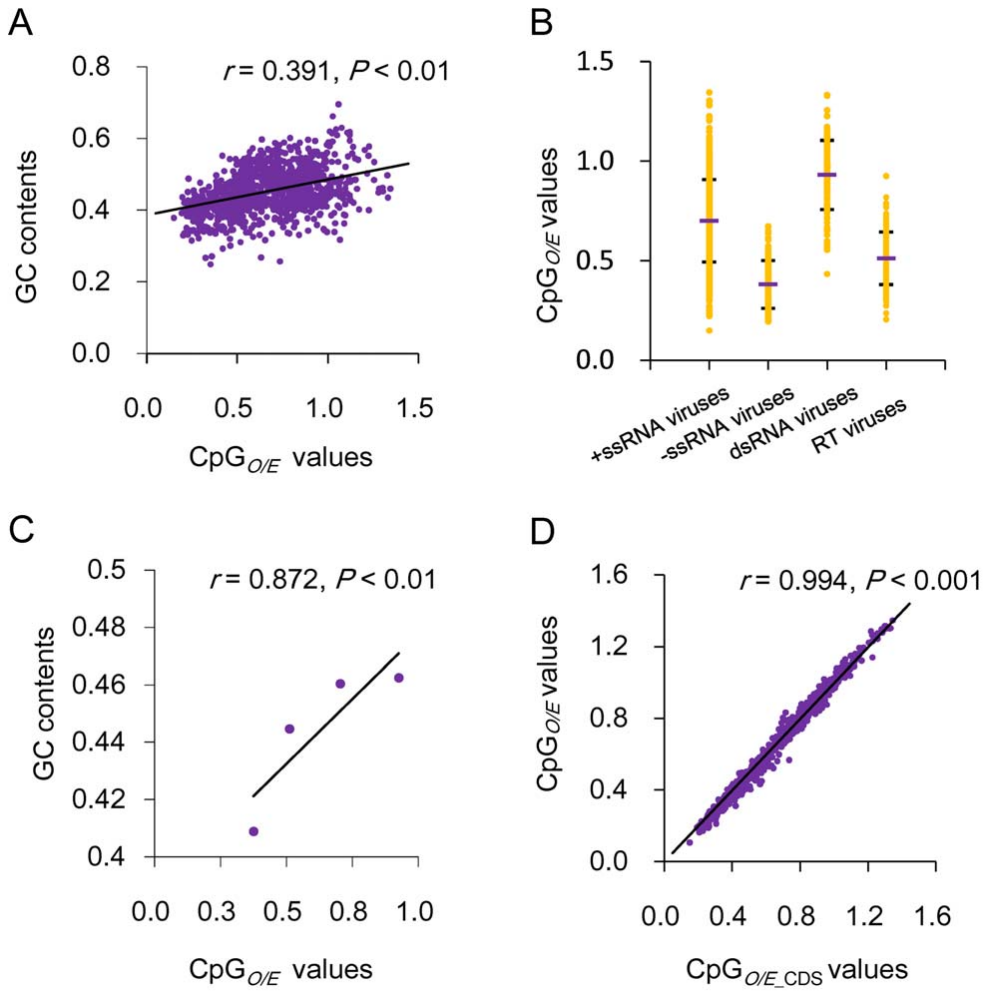
The influence of GC content on viral CpG usage. (**A**) Correlation between CpG odds ratios and GC contents of RNA viruses. (B) CpG usage variation between the four groups of RNA viruses. CpG*_O/E_* distribution range of each viral group is shown by yellow dots, and the mean CpG*_O/E_* value of each viral group is indicated by the purple bar. The standard deviations of the mean CpG*_O/E_* values are also indicated. (**C**) Correlation between the mean CpG*_O/E_* and mean GC content values. (**D**) Correlation between the CpG*_O/E_* and CpG*_O/E_*__CDS_ values.

To understand the possible factors that affect CpG bias in RNA viruses, we first examined the correlation between viral CpG*_O/E_* values and base composition, an important indicator of viral mutational bias [20]. As shown in Figure 2A, the viral genomic G and C content (GC content) positively correlated with their CpG*_O/E_* values (Spearman's rho correlation coefficient *r* = 0.391, *P*<0.01), suggesting that the base composition has a role in shaping the CpG usage of RNA viruses. Previous research has demonstrated that different viral groups may differ from each other in mutation bias [21]. Thus, we suspect that different viral groups may also differ in their CpG usages. Therefore, the 1120 RNA viruses were divided into four groups, namely, double-stranded RNA (dsRNA) viruses, positive single-stranded (+ssRNA) viruses, negative single-stranded (−ssRNA) viruses and RT viruses, which contained 130, 724, 152, and 114 viruses, respectively. As shown in Figure 2B, the −ssRNA viruses have the lowest mean CpG*_O/E_* value (0.38±0.12), followed by RT and +ssRNA viruses (0.51±0.132 and 0.70±0.207), whereas dsRNA viruses have the highest mean CpG*_O/E_* value (0.93±0.174). This is consistent with the mean GC contents of the four viral groups (Table S1). In fact, a strong positive correlation was observed between the mean CpG*_O/E_* and GC content for the four viral groups (Figure 2C). The four viral groups also differed from each other in the distribution range of CpG odds ratio. The −ssRNA viruses have the most convergent distribution range, followed by RT and dsRNA viruses, whereas the +ssRNA viruses have the most divergent CpG distribution range. A variance analysis demonstrated a significant difference in CpG usage between the four groups (Table 2). These results suggest that different viral groups differ in their CpG usage.

**Table 2:** Pair-wised variance analysis CpG usage between viral groups.

	−ssRNA virus	+ssRNA virus	dsRNA virus	RT virus
−ssRNA virus	-	26.049**	30.027**	8.408**
+ssRNA virus		-	12.913**	13.256**
dsRNA virus			-	21.046**
RT virus				-

Note: *F* statistic of one-way ANOVA analysis is 239.252 (*P*<0.001). The pair-wised comparisons were performed based on the CpG*_O/E_* values of each viral group, and the resulting *T* values of the independent *T*-test are shown. ** indicates *P*<0.0001. For the detailed analysis procedure, please refer to the Materials and Methods section.

To further investigate CpG variation between viral families, we calculated the mean CpG*_O/E_* value of each viral family or unassigned genus. The 1120 viruses belong to 61 viral families or unassigned genera, which can be further classed into 9 dsRNA, 37 +ssRNA, 12 −ssRNA, and 3 RT viral families or unassigned genera. CpG was significantly under-represented in all −ssRNA and RT viral families, independent of the phylogenetic relationships of these viral families (Table S2). On the other hand, the viral family or unassigned genus of dsRNA and +ssRNA groups displayed huge variation in CpG usage. For example, the mean CpG*_O/E_* value fluctuated from 0.44 (*Astroviridae*) to 1.16 (*Alphatetraviridae*) in the +ssRNA viral families, and fluctuated from 0.63 (*Birnaviridae*) to 1.18 (*Cystoviridae*) in the dsRNA viral families. Furthermore, +ssRNA viral families in the same order (*Nidovirales*, *Picornavirales*, or *Tymovirales*) also differed from each other greatly in the mean CpG*_O/E_* value (Table S2). These results suggest that there is no obvious relationship between viral CpG content and their phylogeny.

### CpG bias in coding and noncoding regions

The fact that the distribution profile of GpC*_O/E_* which has the same C and G composition as CpG and is regularly used as an indicator of nucleotide composition bias [22], was unimodal (Figures 1 and S1) suggests that there are other factors affecting CpG usage in RNA viruses besides mutational bias. To explore this possibility, we investigated the influence of specific secondary structures and/or base composition in the viral non-coding regions on viral CpG usage. For example, rhinoviruses contains a 3′ U/A rich untranslated region (UTR) that completely lacks CpG [23]. Thus, we calculated the CpG*_O/E_* odds ratios in the coding regions (referred to as CpG*_O/E_*__CDS_) of these RNA viruses (Dataset S3). The genomic CpG*_O/E_* value is very close to the CpG*_O/E_*__CDS_ value of the same RNA virus (Datasets S2 and S3). A correlation analysis indicated that the CpG*_O/E_* values were highly correlated with the CpG*_O/E_*__CDS_ values (Spearman's rho correlation coefficient *r* = 0.994; Figure 2D). These results suggest that biased CpG usage in the non-coding region only has a small influence on overall CpG usage. In other words, the observed under-representation of CpG in RNA viruses is not caused by the biased CpG usage in the non-coding regions but determined mainly by the coding regions.

### Effect of synonymous codon usage

Because CpG usage in RNA viruses is determined mainly by the coding regions, we further analyzed the constraints of synonymous codon usage on CpG usage (Figure 2D). The distribution of CpG in a coding region can be found in three locations, two locations within a codon (CGN or NCG) and one across codon boundaries (the third codon position of the first codon and the first codon position of the following codon). One would expect that if CpG suppression were driven solely by the selection of non CpG-containing synonymous codons, the CpG dinucleotide should be suppressed only within the codons, but not at the location across codon boundaries. However, if CpG were under-represented in all three locations, we could not conclude that CpG bias was under the selection of viral mutational pressure because host-driven CpG elimination pressure may also be involved. Nevertheless, comparing CpG usage at the three locations between a virus and its host may help us to distinguish between host selective pressure and viral mutation bias.

The CpG odds ratios at the three locations of these RNA viruses were calculated separately and designated CpG*_O/E_*__CDS_12_, CpG*_O/E_*__CDS_23_, and CpG*_O/E_*__CDS_31_, respectively (Dataset S3 and Table 1). The mean CpG*_O/E_*__CDS_12_, CpG*_O/E_*__CDS_23_, and CpG*_O/E_*__CDS_31_ values of these RNA viruses were 0.50±0.214, 0.58±0.266, and 0.90±0.395, respectively. Furthermore, 237 viruses were determined to have CpG significantly over-represented at the location across codon boundaries, whereas none or very few viruses (20 viruses) were determined to have CpG significantly over-represented within codons. These results suggest that CpG is consistently under-represented in most RNA viruses at the locations within codons, but varied greatly at the location across codon boundaries.

**Figure 3:**
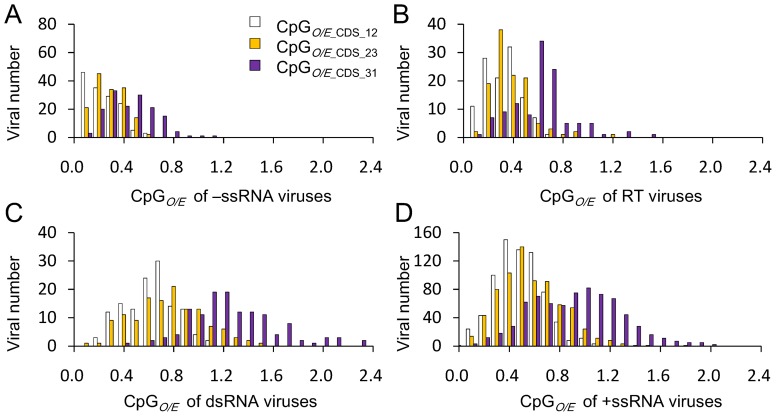
CpG usage pattern of RNA viruses within coding region. (**A–D**) Distribution of CpG at the three locations in the coding regions of −ssRNA, RT, dsRNA, and +ssRNA viruses, respectively.

To gain further insight into the effect of synonymous codon on CpG bias, we further produced CpG distribution profiles covering each location of the four viral groups (Figure 3). In all viral groups, the viral CpG*_O/E_* values at the two locations within codons are apparently lower than the CpG*_O/E_* value at the location across codon boundaries of the same viral groups, suggesting that selection of CpG non-containing synonymous codon usage may be an important evolutionary force driving the CpG usage in RNA viruses. At the location across codon boundaries, CpG was under-represented in most −ssRNA and RT viruses and over-represented in most dsRNA viruses, whereas CpG varied greatly in +ssRNA viruses. Furthermore, the CpG distribution profile of +ssRNA viruses at the position across codon boundaries was bimodal, with one peak representing +ssRNA viruses with under-represented CpG odds ratios and the other peak representing +ssRNA viruses with over-represented CpG odds ratios, suggesting great variation of CpG usage in +ssRNA viruses at the location across codon boundaries.

We further compared CpG usage variation among codons between viral families or unassigned genera (Table S2). Within codons, CpG was under-represented in most viral families or unassigned genera. Conversely, different viral families displayed huge variations in CpG usage at the location across codon boundaries: CpG was significantly under-represented in all −ssRNA and RT viral families and normal or over-represented in all dsRNA viral families, whereas CpG was significantly under-represented in a few +ssRNA viral families and normal to over-represented in other families. These results also suggest that the selection of CpG non-containing synonymous codons may be an important factor affecting viral CpG usage. CpG was consistently under-represented in most −ssRNA and RT viruses but differed greatly among +ssRNA viruses at the location across codon boundaries, which suggests that other factor(s) beside mutation bias and synonymous codon usage may be involved in shaping the CpG usage of RNA viruses.

### Influence of host

Previous studies have shown that the CpG*_O/E_* values of human-infecting riboviruses are lower than that of riboviruses infecting other taxa, suggesting the possible influence of the host on viral CpG usage [14], [18]. To further explore this possibility, the entire referenced mRNA sequence data of 70 species, including 17 species of vertebrates, 14 invertebrates, 9 plants, 12 fungi, and 18 bacteria were downloaded (Dataset S4), and the coding regions were extracted to calculate the CpG odds ratio. In our analysis, the entire mRNA sequence data were used instead of the full genome sequence because host mRNAs are physically present and translated in the cytoplasm where the replication of most RNA viruses takes place. In such a case, the host mRNA should suffer similar selection pressure as the viral RNAs. The coding region was used instead of the entire mRNA sequence to avoid errors induced by the incomplete 5′ and/or 3′ UTRs of some mRNAs and to reduce the possible CpG bias caused by specific RNA structures in host mRNA compared with viral CpG usage. CpG*_O/E_*__CDS_, CpG*_O/E_*__CDS_12_, CpG*_O/E_*__CDS_23_, and CpG*_O/E_*__CDS_ values for each host was calculated. As shown in Dataset S4, the CpG odds ratios of hosts in the same group are very similar, whereas these ratios differed greatly for hosts between groups. These results suggest that hosts in the same group may have similar CpG usage and therefore may exert similar selective pressures on the viruses that infect them. Based on mean CpG*_O/E_*__CDS_ values, it is clear that CpG was under-represented in vertebrates and plants, whereas was normally distributed in fungi, invertebrates, and bacteria (Table 3). In detailed analysis of CpG usage at the three locations separately, it is obvious that different host groups differ greatly in CpG usage, especially at the location across codon boundaries (Table 3). At the location across codon boundaries, CpG was significantly under-represented in vertebrates, normally distributed in plants, whereas it was significantly over-represented in fungi, invertebrates, and bacteria. As mentioned above, CpG usage at this location is independent of synonymous codon usage. Thus, if the hosts have selective pressure on viral CpG usage besides synonymous codon selection, the viruses infecting them should have similar CpG usage at the same location. For instance, if vertebrates have CpG selection pressure on viruses infecting them besides synonymous codon choice, CpG would also be under-represented at the location across codon boundaries of the viruses infecting them. Likewise, CpG should not be under-represented at the location between neighbor codons in fungus-, invertebrate-, and bacterium-infecting RNA viruses.

**Table 3:** CpG usage of RNA viruses and their respective hosts.

	Type^a^	Viral Number	Mean CpG*_O/E_*	Mean CpG*_O/E_*__CDS_12_	Mean CpG*_O/E_*__CDS_23_	Mean CpG*_O/E_*__CDS_31_
Hosts	B	18	1.00±0.167	0.62±0.288	0.93±0.197	1.43±0.409
	F	12	0.88±0.126	0.62±0.072	0.79±0.149	1.24±0.209
	I	14	1.02±0.090	0.60±0.074	1.07±0.163	1.41±0.179
	P	9	0.69±0.206	0.47±0.031	0.64±0.223	0.95±0.372
	V	17	0.47±0.042	0.46±0.176	0.37±0.048	0.58±0.079
+ssRNA viruses	B	9	1.10±0.056	1.00±0.104	0.94±0.118	1.47±0.143
	F	21	0.74±0.186	0.61±0.272	0.63±0.230	0.94±0.409
	I	40	0.87±0.154	0.72±0.162	0.76±0.286	1.10±0.397
	P	432	0.75±1.83	0.54±0.165	0.68±0.232	1.03±0.314
	V	223	0.57±0.183	0.44±0.176	0.46±0.191	0.78±0.339
−ssRNA viruses	I	1	0.57	0.47	0.55	0.71
	P	24	0.35±0.108	0.18±0.086	0.33±0.124	0.49±0.159
	V	126	0.38±0.120	0.28±0.140	0.33±0.122	0.49±0.186
dsRNA viruses	B	3	0.97±0.106	0.58±0.149	0.90±0.265	1.51±0.260
	F	50	0.98±0.174	0.70±0.177	0.80±0.224	1.46±0.420
	I	14	0.91±0.193	0.64±0.257	0.90±0.281	1.21±0.210
	P	26	0.88±0.158	0.67±0.208	0.63±0.237	1.31±0.303
	V	37	0.88±0.168	0.65±0.217	0.88±0.332	1.13±0.250
RT viruses	P	50	0.50±0.126	0.32±0.136	0.38±0.114	0.73±0.236
	V	64	0.52±0.137	0.42±0.124	0.46±0.191	0.59±0.218

a B, indicates bacteria or bacterium-infecting RNA viruses; F, indicates fungi or fungus-infecting RNA viruses; I, indicates invertebrates or invertebrate-infecting RNA viruses; P, indicates plants or plant-infecting RNA viruses; V, indicates vertebrates or vertebrate-infecting RNA viruses.

The host ranges of the 1120 RNA viruses were determined (Dataset S1). Clearly, some RNA viruses can be categorized into two host sets (e.g., many viruses in the family *Flaviviridae* and *Bunyaviridae* can replicate in both invertebrates and vertebrates). These viruses were categorized into the viral group infecting the host class with the lower CpG*_O/E_* value because the host class with the lower CpG*_O/E_* value should exert stronger selection pressure on the viruses that infect them. For example, the arthropod-borne flaviviruses were classified as vertebrate-infecting viruses because the CpG odds ratio of vertebrates is much lower than that of invertebrates [2], [24].

The CpG usage of RNA viruses was compared with that of their respective hosts. CpG was consistently under-represented at the location across codon boundaries in all −ssRNA viruses and RT viruses and over-represented at the same location in all dsRNA viruses, and these results were independent of the host type infected (Table 3). The genomes of vertebrates and plants are highly methylated, and, as a result, the under-representation of CpG in RT viruses may be due to host DNA methylation, because these organisms produce DNA intermediates during the replication of their genome [25], [26]. However, −ssRNA and dsRNA viruses do not produce DNA intermediates during replication and should not be affected by host DNA methylation. Therefore, the consistent under-representation of CpG in −ssRNA viruses and over-representation of CpG in dsRNA viruses at the location between neighboring codons suggest that the CpG usage of −ssRNA and dsRNA viruses at the location may be not deeply affected by their hosts.

**Figure 4:**
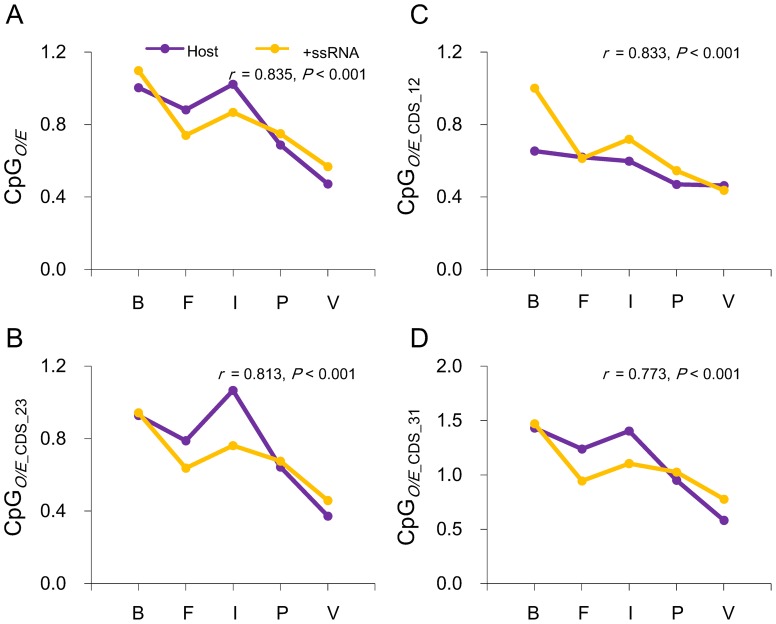
+ssRNA viruses mimic the CpG usage of their respective host. Correlation between +ssRNA viral and host's mean CpG*_O/E_* (**A**), mean CpG*_O/E_*__CDS_12_ (**B**), mean CpG*_O/E_*__CDS_23_ (**C**), and mean CpG*_O/E_*__CDS_31_ (**D**). The abbreviations at the bottom of each chart (B, F, I, P, and V) represent bacteria or bacterial-infecting +ssRNA viruses, fungi or fungus-infecting +ssRNA, invertebrates or invertebrate-infecting +ssRNA viruses, plants or plant-infecting +ssRNA viruses, and vertebrates or vertebrate-infecting +ssRNA viruses, respectively.

Interestingly, CpG was exclusively under-represented in vertebrate +ssRNA viruses, over-represented in bacterial +ssRNA viruses and normally distributed in +ssRNA viruses that infect other host types at the location across codon boundaries, which is consistent with the CpG usage in different host groups at the same location (Table 3). These results indicate that the host may have a role in shaping the CpG usage of +ssRNA viruses. Moreover, CpG usage in +ssRNA viruses within codons was also very similar to that of their respective host. For example, vertebrate +ssRNA viruses have the lowest CpG frequency within codon locations, whereas bacterium +ssRNA viruses have the highest CpG frequency. In fact, the mean CpG*_O/E_* value of +ssRNA viruses at each location was highly correlated with that of its host (Figure 4). Furthermore, within those phylogenetically related +ssRNA viral families (i.e., viral families of the same order), the mean CpG*_O/E_* value of vertebrate-infecting viral families is also significantly lower than that of other viral families that infect other hosts (Table S2). For example, the mean CpG*_O/E_* values of the two vertebrae-infecting families, *Arteriviridae* and *Coronaviridae*, within the order *Nidovirales* are significantly lower than the values of the invertebrate-infecting families within the same order (*Roniviridae*). The CpG*_O/E_* values of the vertebrate-infecting family of the order *Picornavirales* (Picornaviridae) is also significantly lower than other viral families that infect other hosts. Taken together, these results clearly suggest that the host drives the CpG usage in +ssRNA viruses.

Further support for this hypothesis comes from an analysis of viruses from *Flaviviridae* that infect both vertebrate and invertebrate. Previous studies have demonstrated that the overall CpG odds ratio of vertebrate-infecting flaviruses are lower than that of flaviruses that only infect invertebrates, suggesting that the two classes of flaviruses may suffer different CpG selection pressures from their respective host [18], [27]. To further investigate this possibility, we compared the CpG usage of the two groups of flaviruses at the three codon positions separately. The mean CpG odds ratio values of the vertebrate-infecting flaviviruses at the three codon positions were significantly lower than that of flaviviruses that only infect invertebrates (Table 4; pair-wised *t*-test, *P*<0.001). As mentioned above, the CpG usage at the location across codon boundaries is independent of synonymous codon usage or amino acid usage. Thus, differences in CpG bias should arise from host selection instead of mutation bias because viruses of the same family have evolved from the same ancestor and have similar genomic structure and replication mechanisms.

**Table 4:** CpG usages of flaviviruses that infect different types of hosts.

	Invertebrate-infecting flaviviruses	Vertebrate-infecting flaviviruses
Mean CpG*_O/E_*	0.85±0.074	0.50±0.113
Mean CpG*_O/E_*__CDS_	0.87±0.076	0.49±0.117
Mean CpG*_O/E_*__CDS_12_	0.72±0.044	0.33±0.126
Mean CpG*_O/E_*__CDS_23_	0.74±0.100	0.47±0.116
Mean CpG*_O/E_*__CDS_31_	1.16±0.147	0.68±0.178

## Discussion

In this study, we re-examined the CpG frequencies of RNA viruses in an attempt to uncover the mechanism of CpG under-representation in RNA viruses. Our analysis included all available genome sequences of RNA viruses in the RefSeq database, which represents a broader sequence pool than previous studies [8], [9], [14]. Hence, the observations obtained from our dataset are more detailed and more completely reflect the real nature of RNA viruses than previous studies. Our results indicated that CpG under-representation is prevalent in RNA viruses when considering the mean CpG*_O/E_* value (0.67±0.240), which is consistent with earlier observations [8], [9], [14]. Nevertheless, more than 33.6% of the riboviruses (376 of 1,120) analyzed in the present study have normal or over-represented CpG frequencies (CpG*_O/E_*>0.79). This proportion was much higher than in previous studies [8], [9]. This difference is likely caused by the limited number of genomic sequences that were available to previous studies and the subsequently biased data composition; most of the sequences included in previous analyses were human-infecting RNA viruses [8], [9]. Together with the multimodality of the CpG*_O/E_* distribution profiles (Figures 1 and S1), our results clearly demonstrate the existence of huge level of CpG usage diversity in RNA viruses.

Our data revealed that base composition, synonymous codon usage and host selection are the three important factors that affect CpG usage in RNA viruses. Other factors, such as RNA secondary structure in noncoding regions, may also play a minor role in affecting CpG usage in RNA viruses. However, our results indicate that the base composition is the core factor that determines riboviral CpG usage and establishes the “keynote” of riboviral CpG bias. With the exception of bacterium-infecting RNA viruses, all RNA viruses tend to use CpG non-containing synonymous codons, suggesting that the selection of CpG non-containing synonymous codon also plays an important role in shaping CpG usage in RNA viruses. However, our data do not prove that this selection is caused by host driving CpG elimination or amino acid composition bias because base composition also significantly affects viral synonymous codon usage [28], [29], and consistent CpG usage at the locations within codons was only observed between +ssRNA viruses and their respective hosts (Table 3). Based on the analysis of CpG usage at the location across codon boundaries between viral and host groups, host-driving CpG elimination pressure plays an important role in shaping the CpG usage of +ssRNA and RT viruses but not −ssRNA or dsRNA viruses. Taken together, our results indicate that the tremendous variation in CpG usage between −ssRNA, +ssRNA, dsRNA and RT viruses is caused by different combinations of the above mentioned selective pressures, which also differ from each other in strength.

CpG was consistently under-represented in all −ssRNA viruses at all locations within the coding regions. This under-representation of CpG is independent of the infected host or their phylogenetic relationship (Table 3). Together with the fact that −ssRNA viruses are not producing DNA intermediates during the replication of their genome, it is reasonable to believe that the CpG usage of −ssRNA viruses may not be affected greatly by the host they infect. The base composition of all −ssRNA viruses included in our analysis is biased toward UA content (Dataset S1), suggesting that this group of viruses may have a UA mutation bias. A variance analysis demonstrated that the base composition of −ssRNA viruses was significantly different from that of other viral groups (Table 5). These results indicate that −ssRNA viruses have a UA preferred mutation bias, which may result in consistent under-representation of CpG in −ssRNA viruses.

**Table 5:** Pair-wised variance analysis the GC contents between viral groups.

	−ssRNA virus	+ssRNA virus	dsRNA virus	RT virus
−ssRNA viruses	-	11.950**	7.161**	4.517**
+ssRNA viruses		-	0.285	2.257*
dsRNA viruses			-	1.925*
RT virus				-

Note: *F* statistic of one-way ANOVA analysis is 33.343 (*P*<0.001). The pair-wised comparisons were performed based on the GC contents of each viral group and the resulting T values of the independent *T*-test are shown. * indicates *P*<0.05, ** indicates *P*<0.0001.

The most interesting finding of our analysis was that the CpG usage of +ssRNA viruses varied greatly: significantly under-represented in all locations in the +ssRNA viruses infecting vertebrates; under-represented within the codon locations in plants, fungi, and invertebrate +ssRNA viruses; and normal at all locations in bacterium-infecting viruses (Figure 3 and Table 3). Interestingly, this variation was consistent with the infected hosts, which suggest that the host exerts a significant influence on CpG usage in +ssRNA viruses. Vertebrates appear to have the strongest influence on the +ssRNA viruses that infect them, which results in the vertebrate-infecting +ssRNA viruses mimicking vertebrate CpG usage in all coding region locations. Plant-, fungus-, and invertebrate-infecting +ssRNA viruses have CpG under-represented within codons but not at locations between neighboring codons, which suggests that these hosts affect CpG usage of +ssRNA viruses, most likely through synonymous codon selection. This result is consistent with our previous finding that synonymous codon usage by citrus tristeza virus, a woody plant-infecting +ssRNA virus within the *Closteroviridae* family, highly adapts to its citrus host [30]. Bacteria do not present a negative selection toward CpG representation in their genomes [2], [24], [31]. Thus, bacteria may impart no CpG selection pressure on the +ssRNA viruses that infect them. Our results support this conclusion.

In contrast to −ssRNA and +ssRNA viruses, CpG was normal or over-represented in dsRNA viruses at the location across codon boundaries (Tables 3 and S1). Moreover, this characteristic of CpG usage in dsRNA viruses is also independent of the host they infect and their phylogenetic relationship. We believe that this normal frequency usage of CpG in dsRNA viruses is caused by their specific life cycles, in which they produce rare ssRNAs during their genome replication. In eukaryotes, there are two interrelated host antiviral systems, innate immunity and RNA silencing. However, none of the receptors that specifically recognize dsRNA in the two antiviral systems (e. g., Toll-like receptor 3 [TLR3] and RIG-I-like RNA helicases in innate immunity and dsRNA binding protein [DRB] in RNA silencing) display any CpG preference [32]–[35]. Consequently, CpG elimination pressure from the host perhaps cannot act on dsRNA viruses.

RT viruses represent a special group of RNA viruses in our analysis, because they produce DNA intermediates during their genome replication. DNA methylation has been shown to play an important role in the antiviral response against RT viruses and endogenous retrotransposons [25], [26], [36]. These results are consistent with the consistent under-representation of CpG in RT viruses, especially at the location across codon boundaries. Interestingly, the CpG odds ratios of the vertebrate-infecting RT viruses were also significantly lower than that of plant-infecting RT viruses, suggesting that in addition to cytosine methylation, there is other CpG selection pressure from vertebrates acting on the RT viruses to infect them. In other words, vertebrate-infecting RT viruses may suffer two types of CpG selection pressures from their host, one most likely through DNA methylation, and the other may be the same as the CpG elimination pressure that acts on +ssRNA viruses at the RNA level.

Based on our results, host driving of CpG elimination at the RNA level appears unique to vertebrates. Studies have shown that ssRNAs with specific sequence motifs (e.g., motifs of AU- or GU-rich and CpG flanked by AU) can significantly stimulate the antiviral immune responses by promoting the secretion of type I interferon [15], [16]. Furthermore, exogenous or abnormal ssRNA is believed to be detected by toll-like receptors (TLRs), such as toll-like receptor 7/8 (TLR7/8) in mammal cells [37]–[40]. However, there is no direct clue to support the idea that host driving of CpG elimination at the RNA level is dived directly by TLRs, such as TLR7/8. In addition, TLRs and TLR-like proteins have been found in almost all eukaryotic organisms, including vertebrates, insects and plants [32], [33], [41]–[44]. Thus, further research is needed to uncover the mechanism of CpG elimination in RNA viruses.

## Methods

### Data acquisition and treatment

All viral sequence data were downloaded from the RefSeq database available at the National Center for Biotechnology Information (NCBI) website (http://www.ncbi.nlm.nih.gov) in GenBank format (accessed on Mar. 05, 2013). The genomic sequences of RNA viruses were identified using a BioPython script, which also filtered inaccurate sequences (containing ambiguous nucleotides, ORFs not in a multiple of three, or non-valid start and/or stop codons). The final dataset contained 1,955 sequences, which represented the full genome of 1120 RNA viruses.

All reference mRNA sequences of viral hosts were either directly downloaded from the NCBI RefSeq database using the taxonomic name of the host species as the query, the molecular type limited to mRNA, and the gene location limited to the nucleus or fetched from complete genome sequences downloaded from GenBank using a BioPython script. Each data set was then applied to a BioPython pipeline to extract the open reading frames (ORFs) and remove any erroneous sequences using the same conditions. Sequences containing ambiguous nucleotides or fewer than 300 nucleotides were excluded from our analysis. The final complete mRNA sequence set for all hosts can be provided upon request.

### Dinucleotide odds ratio analysis

The dinucleotide odds ratio was defined as the observed frequency of a dinucleotide pair in a given sequence divided by the frequencies of the two mononucleotides that form the dinucleotide pair in the same sequence. The dinucleotide odds ratio for RNA viruses was calculated based on the following equation:
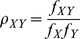
where *f_X_* and *f_Y_* denote the frequencies of the mononucleotides *_X_* and *_Y_* in a given sequence, and *f_XY_* denotes the frequency of dinucleotide *_XY_* in the same sequence. Using statistical theory, the dinucleotide relative abundance may be conservatively described as significantly low if *ρ_XY_* ≤0.78 and significantly high if *ρ_XY_* ≥1.23 [5].

In the case of double-stranded DNA (dsDNA), the frequency of each dinucleotide must be calculated in a symmetric manner considering the complementary sequence. Thus, a symmetric version of the dinucleotide odds ratio, ρ*_XY_, can be calculated based on the following equation:
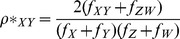
where *_X_* and *_Y_* denote the two dinucleotides, and *_Z_* and *_W_* indicate the two complementary nucleotides of *_Y_* and *_X_*, respectively.

All calculations were accomplished using BioPython scripts that are available upon request.

### Viral hosts range mapping

The viral host range was determined based on the information provided with the sequence file, from the Ninth Report of the International Committee on Taxonomy of Viruses, ViralZone database (http://viralzone.expasy.org/), or Wikipedia (http://en.wikipedia.org/). However, for some viruses, the host ranges were verified by a literature search.

### Statistical analysis

Correlation analyses were performed using SPSS (Statistical Package for the Social Sciences) 16.0 software (SPSS Inc., Chicago, Illinois, USA). For variance analyses of CpG*_O/E_* values and GC content between the different viral groups, we first performed one-way analysis of variance (one-way ANOVA) F-tests using Fisher's Least Significant Difference (LSD) method. There were significant differences between different viral groups when the resulting *P* value was <0.05. Additionally, the pair-wised independent *t*-tests were performed to analyze whether the viral groups were significantly different from one another. All variance analyses were performed using SPSS 16.0 software.

## Supporting Information

Figure S1 **Dinucleotide usage patterns of RNA viruses.** The *y*-axis depicts the number of viruses with the specific CpG*_O/E_* values given on the *x*-axis. (A–O) Distribution patterns of ApA, ApT, ApG, ApC, TpA, TpT, TpG, TpC, CpA, CpT, CpC, GpA, GpT, GpG and GpC, respectively. Note that the distribution pattern of TpA negatively deviates from the normal frequency range (0.79–1.22), whereas the distribution patterns of TpG and CpA positively deviate from the normal frequency range, suggesting TpA was under-represented in most RNA viruses and TpG and CpA were over-represented in most RNA viruses.
(PDF)

Table S1 **Mean GC content of different viral groups.**
(DOCX)

Table S2 **CpG usage variation between different viral families.**
(DOCX)

Dataset S1 **RNA virus information.**
(XLSX)

Dataset S2 **Genomic dinucleotide odd ratio values and base compositions of RNA viruses.**
(XLSX)

Dataset S3 **CpG odd ratio values of coding regions of RNA viruses.**
(XLSX)

Dataset S4 **CpG odd ratio values of hosts.**
(XLSX)
