# Supplementary figures and images for: CpG Usage in RNA Viruses: Data and Hypotheses
Source: PLoS One. 2013 Sep 23;8(9):e74109. doi: 10.1371/journal.pone.0074109 (PMC3781069; doi:10.1371/journal.pone.0074109)

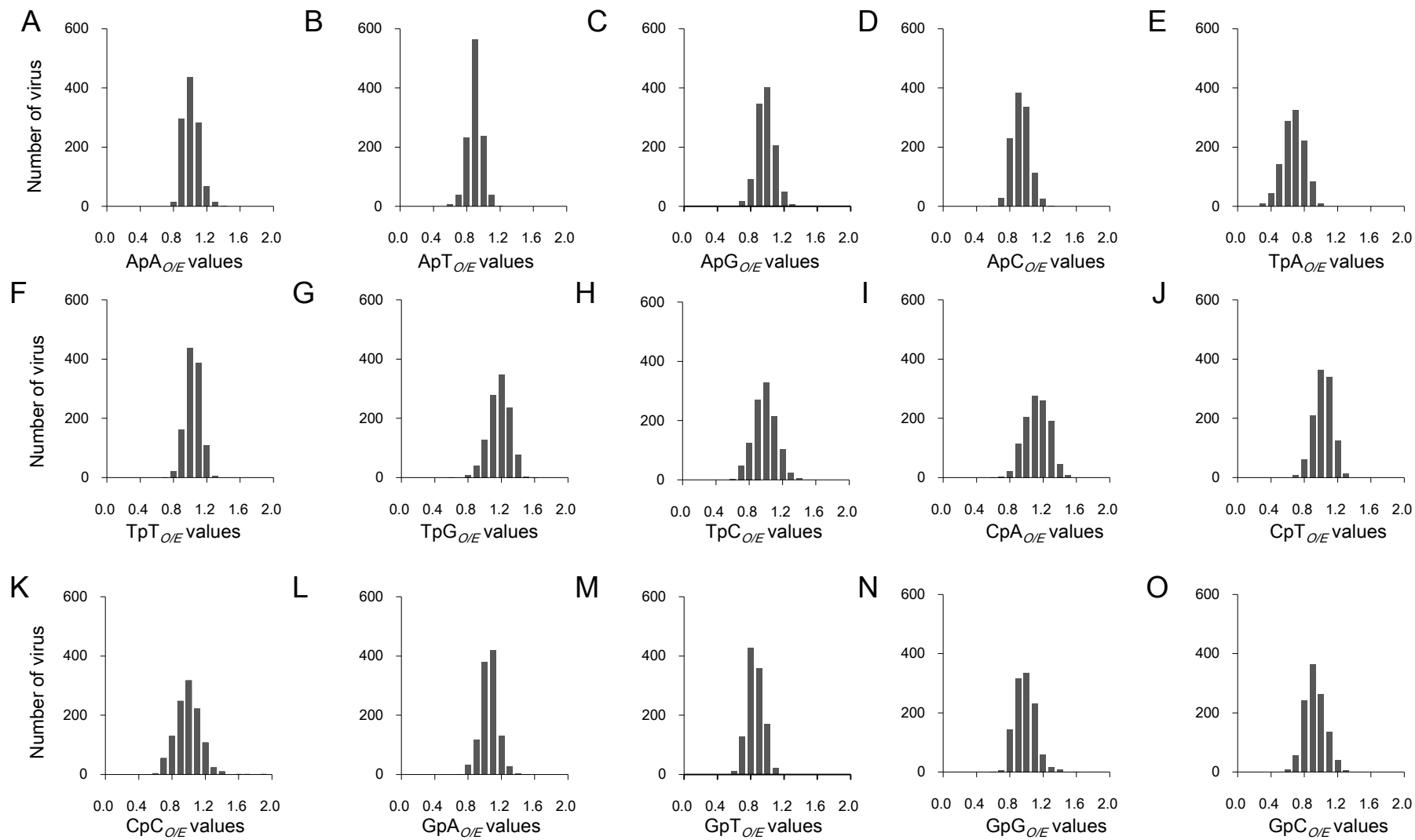

Supplement: Figure S1 — Dinucleotide usage patterns of RNA viruses. The y-axis depicts the number of viruses with the specific CpGO/E values given on the x-axis. (A–O) Distribution patterns of ApA, ApT, ApG, ApC, TpA, TpT, TpG, TpC, CpA, CpT, CpC, GpA, GpT, GpG and GpC, respectively. Note that the distribution pattern of TpA negatively deviates from the normal frequency range (0.79–1.22), whereas the distribution patterns of TpG and CpA positively deviate from the normal frequency range, suggesting TpA was under-represented in most RNA viruses and TpG and CpA were over-represented in most RNA viruses. (PDF) [file pone.0074109.s001.pdf]
