# Supplementary material for: CpG Usage in RNA Viruses: Data and Hypotheses
Source: PLoS One. 2013 Sep 23;8(9):e74109. doi: 10.1371/journal.pone.0074109 (PMC3781069; doi:10.1371/journal.pone.0074109)
Supplement: Table S1 — Mean GC content of different viral groups. (DOCX) [file pone.0074109.s002.docx]

**Table S1 Mean GC content of different viral groups**

| Viral group | Mean GC content |
| --- | --- |
| –ssRNA viruses | 0.41 ± 0.045 |
| +ssRNA viruses | 0.46 ± 0.056 |
| dsRNA viruses | 0.46 ± 0.072 |
| Retroviruses | 0.44 ± 0.071 |
