# Supplementary material for: CpG Usage in RNA Viruses: Data and Hypotheses
Source: PLoS One. 2013 Sep 23;8(9):e74109. doi: 10.1371/journal.pone.0074109 (PMC3781069; doi:10.1371/journal.pone.0074109)
Supplement: Table S2 — CpG usage variation between different viral families. (DOCX) [file pone.0074109.s003.docx]

**Table S2** **CpG usage variation between different viral families**

| Order | Family | Genome polarity*^a^* | Host Type*^b^* | Number | CpG*_O/E_* | CpG*_O/E_*__CDS_12_ | CpG*_O/E_*__CDS_23_ | CpG*_O/E_*__CDS_31_ |
| --- | --- | --- | --- | --- | --- | --- | --- | --- |
| Mononegavirales | Bornaviridae | (-) | V | 1 | 0.65 | 0.58 | 0.64 | 0.74 |
| Mononegavirales | Filoviridae | (-) | V | 7 | 0.54±0.04 | 0.57±0.06 | 0.47±0.06 | 0.58±0.09 |
| Mononegavirales | Paramyxoviridae | (-) | V | 35 | 0.43±0.11 | 0.33±0.10 | 0.39±0.12 | 0.56±0.16 |
| Mononegavirales | Rhabdoviridae | (-) | V,I,P | 26 | 0.47±0.09 | 0.32±0.08 | 0.43±0.08 | 0.66±0.17 |
| - | Arenaviridae | (-) | V | 30 | 0.27±0.03 | 0.13±0.04 | 0.23±0.05 | 0.35±0.06 |
| - | Bunyaviridae | (-) | V,P | 35 | 0.29±0.07 | 0.25±0.13 | 0.25±0.07 | 0.37±0.12 |
| - | Ophioviridae | (-) | P | 3 | 0.41±0.08 | 0.18±0.08 | 0.39±0.06 | 0.53±0.16 |
| - | Orthomyxoviridae | (-) | V | 9 | 0.41±0.07 | 0.28±0.11 | 0.36±0.06 | 0.63±0.12 |
| - | Deltavirus | (-) | V | 1 | 0.71 | 0.59 | 0.67 | 0.86 |
| - | Emaravirus | (-) | P | 2 | 0.27±0.03 | 0.24±0.08 | 0.18±0.07 | 0.29±0.10 |
| - | Tenuivirus | (-) | P | 2 | 0.23±0.01 | 0.10±0.00 | 0.20±0.05 | 0.33±0.01 |
| - | Varicosavirus | (-) | P | 1 | 0.54 | 0.23 | 0.44 | 0.81 |
| Nidovirales | Arteriviridae | (+) | V | 4 | 0.69±0.06 | 0.54±0.05 | 0.56±0.07 | 0.98±0.13 |
| Nidovirales | Coronaviridae | (+) | V | 36 | 0.51±0.06 | 0.53±0.08 | 0.36±0.11 | 0.62±0.18 |
| Nidovirales | Roniviridae | (+) | I | 1 | 0.87 | 0.64 | 0.31 | 1.67 |
| Picornavirales | Dicistroviridae | (+) | I | 12 | 0.77±0.17 | 0.73±0.20 | 0.67±0.20 | 0.88±0.28 |
| Picornavirales | Iflaviridae | (+) | I | 8 | 0.84±0.11 | 0.73±0.21 | 0.96±0.45 | 0.80±0.11 |
| Picornavirales | Marnaviridae | (+) | P | 1 | 0.95±0.94 | 0.82±0.79 | 0.92±0.91 | 1.12±1.14 |
| Picornavirales | Picornaviridae | (+) | V | 66 | 0.53±0.19 | 0.36±0.15 | 0.38±0.14 | 0.76±0.41 |
| Picornavirales | Secoviridae | (+) | P | 36 | 0.56±0.12 | 0.55±0.15 | 0.48±0.13 | 0.60±0.19 |
| Tymovirales | Alphaflexiviridae | (+) | P | 36 | 0.71±0.17 | 0.41±0.13 | 0.56±0.15 | 1.15±0.33 |
| Tymovirales | Betaflexiviridae | (+) | P | 48 | 0.58±0.18 | 0.40±0.17 | 0.48±0.16 | 0.84±0.27 |
| Tymovirales | Gammaflexiviridae | (+) | F | 1 | 0.85 | 0.58 | 0.49 | 1.56 |
| Tymovirales | Tymoviridae | (+) | P | 26 | 0.81±0.15 | 0.68±0.09 | 0.47±0.17 | 1.28±0.34 |
| - | Astroviridae | (+) | V | 19 | 0.44±0.10 | 0.50±0.12 | 0.32±0.13 | 0.50±0.18 |
| - | Barnaviridae | (+) | F | 1 | 0.94 | 0.73 | 1.02 | 1.09 |
| - | Bromoviridae | (+) | P | 30 | 0.92±0.09 | 0.64±0.11 | 0.88±0.15 | 1.27±0.19 |
| - | Caliciviridae | (+) | V | 20 | 0.63±0.10 | 0.45±0.08 | 0.50±0.09 | 0.94±0.24 |
| - | Closteroviridae | (+) | P | 29 | 0.91±0.24 | 0.47±0.21 | 0.91±0.24 | 1.34±0.36 |
| - | Flaviviridae | (+) | V,I | 54 | 0.53±0.15 | 0.37±0.17 | 0.49±0.14 | 0.72±0.22 |
| - | Hepeviridae | (+) | V | 3 | 0.69±0.12 | 0.63±0.19 | 0.48±0.16 | 0.96±0.19 |
| - | Hypoviridae | (+) | F | 5 | 0.74±0.12 | 0.62±0.10 | 0.61±0.11 | 0.94±0.23 |
| - | Leviviridae | (+) | B,F | 10 | 1.07±0.10 | 0.96±0.15 | 0.93±0.12 | 1.44±0.17 |
| - | Luteoviridae | (+) | P | 21 | 0.83±0.08 | 0.62±0.09 | 0.71±0.14 | 1.20±0.14 |
| - | Narnaviridae | (+) | F | 11 | 0.67±0.21 | 0.61±0.38 | 0.62±0.28 | 0.75±0.34 |
| - | Nodaviridae | (+) | V,I | 14 | 0.95±0.08 | 0.87±0.19 | 0.69±0.18 | 1.29±0.19 |
| - | Potyviridae | (+) | P | 99 | 0.70±0.14 | 0.61±0.13 | 0.63±0.14 | 0.88±0.23 |
| - | Alphatetraviridae | (+) | I | 3 | 1.16±0.04 | 0.72±0.12 | 0.98±0.06 | 1.82±0.11 |
| - | Carmotetraviridae | (+) | I | 1 | 0.8 | 0.63 | 0.72 | 1.04 |
| - | Permutotetraviridae | (+) | I | 1 | 0.8 | 0.69 | 0.94 | 0.73 |
| - | Togaviridae | (+) | V | 19 | 0.88±0.08 | 0.45±0.10 | 0.88±0.11 | 1.27±0.22 |
| - | Tombusviridae | (+) | P | 40 | 0.75±0.08 | 0.58±0.12 | 0.71±0.15 | 1.02±0.17 |
| - | Virgaviridae | (+) | P | 42 | 0.93±0.09 | 0.48±0.14 | 1.02±0.17 | 1.25±0.17 |
| - | Benyvirus | (+) | P | 2 | 0.83±0.04 | 0.81±0.03 | 0.59±0.18 | 1.01±0.04 |
| - | Idaeovirus | (+) | P | 1 | 0.82 | 0.48 | 0.72 | 1.32 |
| - | Ourmiavirus | (+) | P | 3 | 0.90±0.04 | 0.89±0.06 | 0.76±0.01 | 1.09±0.09 |
| - | Polemovirus | (+) | P | 1 | 0.8 | 0.71 | 0.71 | 1.01 |
| - | Sobemovirus | (+) | P | 15 | 0.77±0.07 | 0.49±0.08 | 0.75±0.12 | 1.03±0.12 |
| - | Umbravirus | (+) | P | 5 | 0.72±0.04 | 0.61±0.07 | 0.60±0.07 | 0.95±0.10 |
| - | Birnaviridae | dsRNA | V,I | 8 | 0.63±0.04 | 0.36±0.07 | 0.45±0.11 | 1.06±0.13 |
| - | Chrysoviridae | dsRNA | F | 4 | 0.82±0.16 | 0.45±0.16 | 0.89±0.22 | 1.08±0.23 |
| - | Cystoviridae | dsRNA | F | 5 | 1.18±0.06 | 0.64±0.03 | 1.08±0.06 | 1.88±0.15 |
| - | Endornaviridae | dsRNA | P,F | 8 | 0.65±0.14 | 0.41±0.10 | 0.55±0.15 | 1.00±0.22 |
| - | Partitiviridae | dsRNA | F,P | 30 | 0.99±0.15 | 0.81±0.14 | 0.58±0.20 | 1.57±0.42 |
| - | Megabirnavirus | dsRNA | F | 1 | 0.94 | 0.79 | 0.91 | 1.14 |
| - | Picobirnaviridae | dsRNA | V | 1 | 0.8 | 0.78 | 0.64 | 0.99 |
| - | Reoviridae | dsRNA | V,I,P,F | 48 | 0.97±0.13 | 0.73±0.18 | 0.97±0.26 | 1.21±0.26 |
| - | Totiviridae | dsRNA | F,P,I,V,B | 25 | 0.92±0.13 | 0.60±0.16 | 0.85±0.16 | 1.33±0.29 |
| - | Caulimoviridae | RT | P | 50 | 0.50±0.13 | 0.32±0.14 | 0.38±0.11 | 0.73±0.24 |
| - | Hepadnaviridae | RT | V | 9 | 0.55±0.06 | 0.52±0.07 | 0.54±0.06 | 0.67±0.11 |
| - | Retroviridae | RT | V | 55 | 0.51±0.15 | 0.40±0.12 | 0.45±0.20 | 0.58±0.23 |
|  |  |  |  |  |  |  |  |  |
| *^a^*(-), -ssRNA viruses; (+), +ssRNA viruses; dsRNA, dsRNA viruses; RT, reverse-transcribing viruses | | | | | |  |  |  |
| *^b^*B, bacteria-infecting; F, fungium infecting; I, invertebrate-infecting; P, plant-infecting; V, vertebrate-infecting. | | | | | |  |  |  |
